# Lectin-Based Assay for Glycoform-Specific Detection of α2,6-sialylated Transferrin and Carcinoembryonic Antigen in Tissue and Body Fluid

**DOI:** 10.3390/molecules23061314

**Published:** 2018-05-30

**Authors:** Hiromi Ito, Kyoka Hoshi, Takashi Honda, Yasuhiro Hashimoto

**Affiliations:** 1 Department of Biochemistry, Fukushima Medical University School of Medicine, Fukushima 960-1295, Japan; itohrm@fmu.ac.jp (H.I.); khoshi@fmu.ac.jp (K.H.); 2 Devision of Human Life Science, Fukushima Medical University School of Nursing, Fukushima 960-1295, Japan; ponchan@fmu.ac.jp

**Keywords:** adenocarcinoma, antigen-antibody reaction, automated latex-agglutination immunoassay, carcinoembryonic antigen, cerebrospinal fluid, immunohistochemistry, enzyme-linked immunosorbent assay, *Psathyrella velutina lectin*, *Sambucus sieboldiana* agglutinin, transferrin, sialylα2,6galactose

## Abstract

Antibodies are useful for detecting glycoprotein antigens, but a conventional antibody recognizes only a protein epitope rather than a glycan. Thus, glycan isoform detection generally requires time- and labor-consuming processes such as lectin affinity column chromatography followed by sandwich ELISA. We recently found antigen-antibody reactions that were inhibited by lectin binding to glycans on the glycoprotein antigen, leading to a convenient glycoform-specific assay. Indeed, *Sambucus sieboldiana* agglutinin (SSA) lectin, a binder to sialylα2,6galactose residue, inhibited antibody binding to α2,6-sialylated transferrin (Tf) (SSA inhibition). SSA inhibition was not observed with other glycoforms, such as periodate-treated, sialidase-treated and sialidase/galactosidase-treated Tf, suggesting that the assay was glycoform-specific. SSA inhibition was also applicable for visualizing localization of α2,6-sialylated-Tf in a liver section. This is the first immunohistochemical demonstration of glycoform localization in a tissue section. SSA inhibition was utilized for establishing ELISA to quantify α2,6-sialylated carcinoembryonic antigen (CEA), a marker for various cancers. In addition, α2,6-sialylated-CEA was visualized in a colonic adenocarcinoma section by SSA inhibition. The method would further be applicable to a simple and rapid estimation of other α2,6-sialylated glycoproteins and have a potential aid to histopathological diagnosis.

## 1. Introduction

Glycosylation is the most extensive and complex post-translational modification and affect properties of the proteins and lipids to which glycan attach. Alteration of glycan’s structure and its site-specificity modulates biological phenotypes such as development, immunity, and disease susceptibility. Abnormal glycosylation is detected in various pathological processes; malignant transformation and immune abnormalities. Accordingly, glycoproteomics is drawing great attention as an emerging research field, along with genomics and proteomics [1,2]. It is a challenge to clarify complex glycan structures of glycoconjugates, which may have a large variety of isomeric variations such as anomer, diastereomer, positional isomer, including multi-antennary structure, or repeating units [2,3,4]. These glycan structures have been analyzed by high-performance liquid chromatography (HPLC), capillary electrophoresis (CE), mass spectrometry (MS), nuclear magnetic resonance (NMR), and various combinations thereof. Such methods provide structural information in detail but they need multiple purification steps in conjunction with the analyses.

Lectins are carbohydrate-binding proteins that specifically recognize glycan structures and have been utilized for profiling glycoproteins in crude specimen [5,6,7]. For example, a microarray immobilizing 45 kinds of lectins identified unique glycan changes on Mac-2 binding protein, which is a serum marker for hepatic fibrosis [8]. Lectins can also detect glycoforms captured on a microtiter plate, i.e., lectin-probed enzyme-linked immunosorbent assay (ELISA) [9]. We have recently developed another ELISA-based glycoform detection method, in which a lectin inhibits antigen-antibody binding when the antigen carries lectin epitope(s) (lectin inhibition) [10,11,12]. Our lectin inhibition assay is useful for rapid and specific detection of a particular glycoform of an antigen. Here we summarize the application of lectin inhibition in sandwich ELISA, automated latex-agglutination immunoassay, and immunohistochemistry.

## 2. Lectins Inhibits Anti-Transferrin Antibody Binding to the Antigen in ELISA

**Figure 1:**
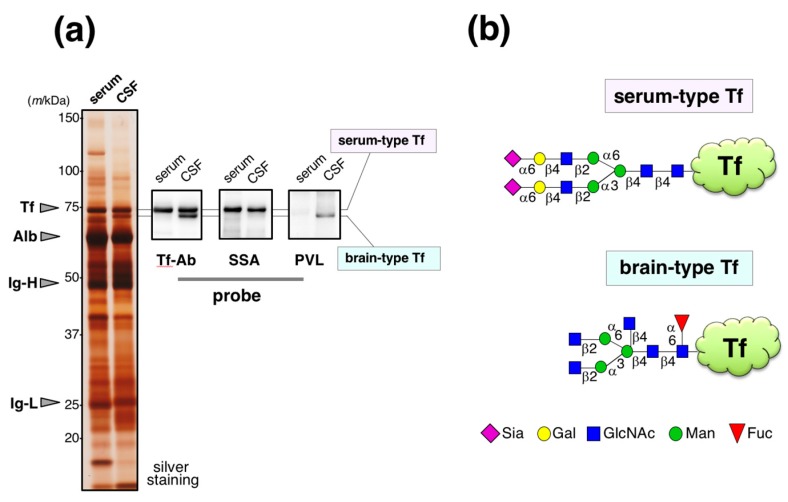
Glycoforms of Tf in CSF. (**a**) CSF and serum are analyzed by SDS-PAGE followed by silver staining. Tf-Ab (Bethyl) was used for Western blotting. SSA and PVL were used for lectin blotting. Migrating positions of purified serum Tf (Tf), albumin (Alb), immunoglobulin heavy chain (Ig-H), and light chain (Ig-L) are indicated with arrowheads (Hoshi et al., unpublished data). In CSF, the upper and lower bands are designated as serum-type and brain-type Tf, respectively; (**b**) Their schematic glycan structures are illustrated [15]. The Figure 1a data is adapted with permission from Murakami et al., *J. Biochem.*, Oxford University Press, *in press*.

Human serum and cerebrospinal fluid (CSF) were analyzed by SDS-PAGE followed by silver staining. CSF band patterns of major proteins are similar to those in serum (Figure 1a). Indeed, 80% of CSF was derived from serum [13]. The specimens were also analyzed by Western blotting using anti-Tf antibody (Ab) from Bethyl Laboratories. Serum Tf is detected as a single band around 74 kDa. In CSF, two bands were detected; one shows similar migration position with serum Tf and the other does not. *Sambucus sieboldiana* agglutinin (SSA) lectin blotting reveals that the former binds to SSA as serum Tf, indicating both have α2,6-sialic acid residues. In contrast, *Psathyrella velutina lectin* (PVL) binds the latter, but not the former, indicating the presence of *N*-acetylglucosamine (GlcNAc) residue(s) on its glycans. The former was designated as serum-type Tf and the latter was brain-type Tf. Each of the glycoforms were purified and their glycan structures were analyzed by authentic analytical methods including mass spectrometry. We found that serum-type Tf has complex-type biantennary *N*-glycans carrying a sialylα2,6galactose sequence, which is a binding epitope for SSA lectin, and brain-type Tf has complex-type biantenary *N*-glycans carrying bisecting GlcNAc and core-fucose (Figure 1b) [14,15].

**Figure 2:**
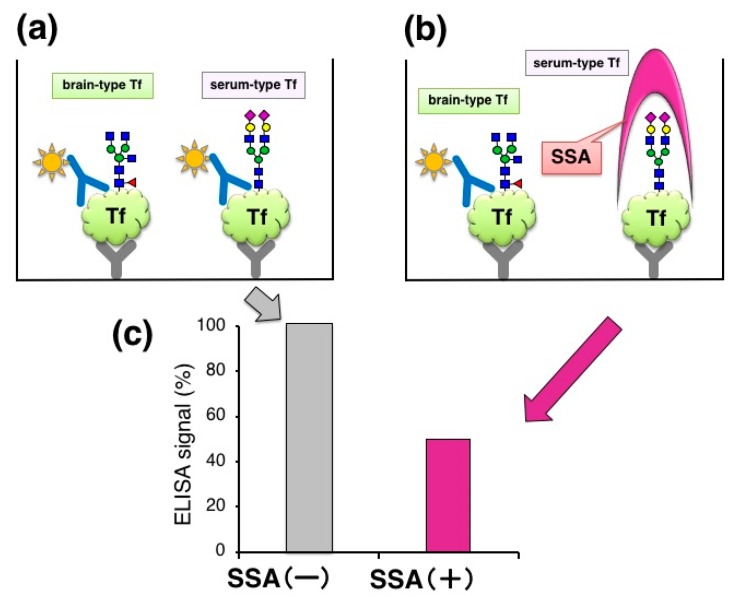
Lectin inhibition of ELISA signal. (**a**) Quantification of Tf in CSF by sandwich ELISA; (**b**) Addition of SSA lectin in the ELISA system; (**c**) ELISA signals with and without SSA lectin. Signals (%) relative to control (+BSA) are indicated.

We also found that serum-type/brain-type Tf ratio was a diagnostic marker for idiopathic normal pressure hydrocephalus (iNPH), a senile dementia, which is caused by abnormal CSF metabolism [15]. The marker change was mainly due to a decrease of brain-type Tf, a unique component in CSF. For clinical use, the ratio was analyzed by Western blotting as a standard method. Total Tf was estimated by Tf-Ab/Tf-Ab sandwich ELISA (Figure 2a). We tried to add SSA lectin, a strong binder to α2,6-sialic acid, in ELISA with the expectation that SSA may compete with Tf-Ab, leading to glycoforms specific assay. For SSA inhibition assay, the capture Tf-Ab (goat polyclonal, Cappel; ICN Pharmaceuticals, Aurora, OH, USA) was pre-treated with sodium periodate (NaIO_4_) to destroy SSA epitopes on the antibody. Periodate treatment did not affect Ab binding to antigens (data not shown). The detection antibody was HRP-conjugated anti-human Tf-Ab (goat polyclonal, Bethyl laboratories, Montgomery, TX, USA). We found that addition of SSA lectin (J-Oil Mills, Kanagawa, Japan) significantly attenuates the ELISA signal (Figure 2b,c) [10]. This suggests that SSA inhibits antigen-antibody reactions by binding to α2,6-sialyl residues on serum-type Tf (lectin inhibition).

**Figure 3:**
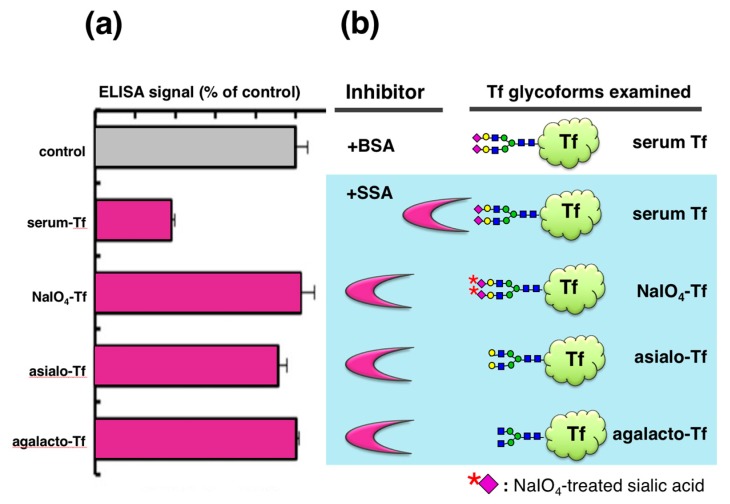
Lectin inhibition with various glycoforms of Tf. Their ELISA signals in the presence of SSA are expressed as % of control (+BSA) (**a**). Tf glycoforms used are serum Tf, NaIO_4_-Tf, asialo-Tf, and agalacto-Tf (**b**). The Figure 3a data is adapted with permission from Hoshi et al., *J. Biochem.*, Oxford University Press, 2013 [10].

To analyze the glycan specificity of lectin inhibition, we prepared a series of Tf glycoforms having different non-reducing terminal sugars (Figure 3). Serum Tf, having the same *N*-glycan as serum-type Tf in CSF, was treated with 1 mM NaIO_4_ to oxidize the exocyclic glycerol side chain of sialic acid, abolishing binding to SSA. Serum Tf was also treated with sialidase to generate asialo-Tf. Asialo-Tf was further digested with β-galactosidase to generate agalacto-Tf. Tf glycoforms so prepared were subjected to lectin inhibition assay. SSA attenuates the ELISA signal of serum Tf but not those of other glycoforms, indicating that lectin inhibition is glycoform- or glycan-specific (Figure 3a).

SSA inhibits Tf signal in a dose-dependent manner. The signal (OD 1.0) decreases linearly in the presence of 0.2–2 μg/mL of SSA and then saturates in a range of 10–30 μg/mL. The maximum rate of inhibition under the aforementioned experimental conditions is approximately 60% [10]. By ELISA, we tested three kinds of antibodies in an SSA inhibition assay: goat polyclonal Tf-Ab from Cappel, rabbit polyclonal Tf-Ab from DAKO Co., Ltd. (Glostrup, Denmark), and goat polyclonal Tf-Ab from Bethyl Laboratories. Their binding to serum Tf was inhibited by SSA: ca. 50–60% inhibition for Cappel antibody and ca. 30% inhibition for DAKO and Bethyl antibodies [10]. The three polyclonal antibodies were more or less inhibited by SSA.

**Figure 4:**
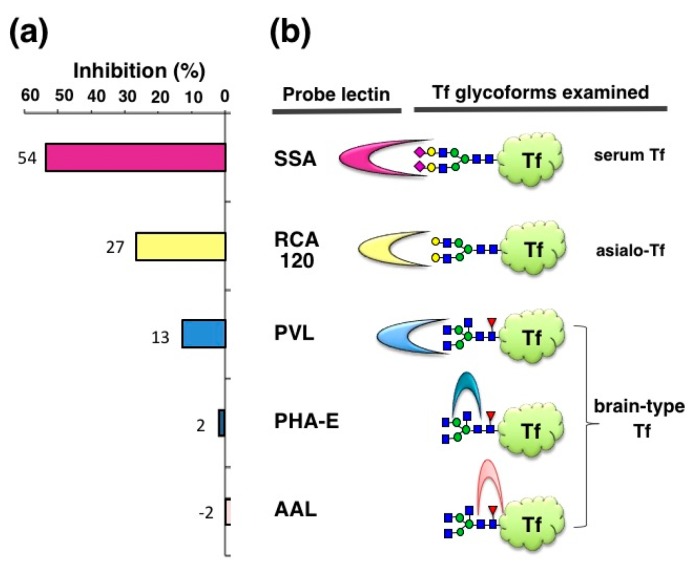
Inhibition of ELISA signals by various lectins. Inhibitory potencies of various lectins are examined with purified Tf glycoforms: (**a**) Inhibitions (%) relative to control (+BSA) are indicated (Hoshi et al., unpublished data); (**b**) SSA for serum Tf, RCA120 for sialo-Tf, PVL for brain-type Tf, PHA-E for brain-type Tf, and AAL for brain-type Tf were used.

We also examined inhibitory potencies of other lectins—RCA120, PVL, PHA-E, and AAL (120–1250 μg/mL)—with the expectation that some inhibitors for brain-type Tf would be useful for iNPH diagnosis. RCA120 binds terminal Gal residues, which are carried by asialo-Tf. RCA120 inhibits asialo-Tf signal by 27% in ELISA (Figure 4). PVL is a binder to terminal GlcNAc residue, which is carried by brain-type Tf and agalacto-Tf. PVL inhibits brain-type Tf signal by 13%. It is notable that brain-type Tf has bisecting GlcNAc and core fucose in addition to its agalacto-structure. PHA-E and AAL are binders to bisecting GlcNAc and core fucose, respectively. These lectins barely inhibit brain-type Tf signal in ELISA. These results suggest that some lectins other than SSA inhibit antigen-antibody binding, but their inhibitory potencies are much less than SSA (Hoshi et al., unpublished data). Thus, SSA lectin is the most potent inhibitor for anti-Tf antibody binding to α2,6-sialylated Tf and “SSA inhibition” is further characterized as described below.

## 3. Application of SSA Inhibition to Auto-Analyzer Method

**Figure 5:**
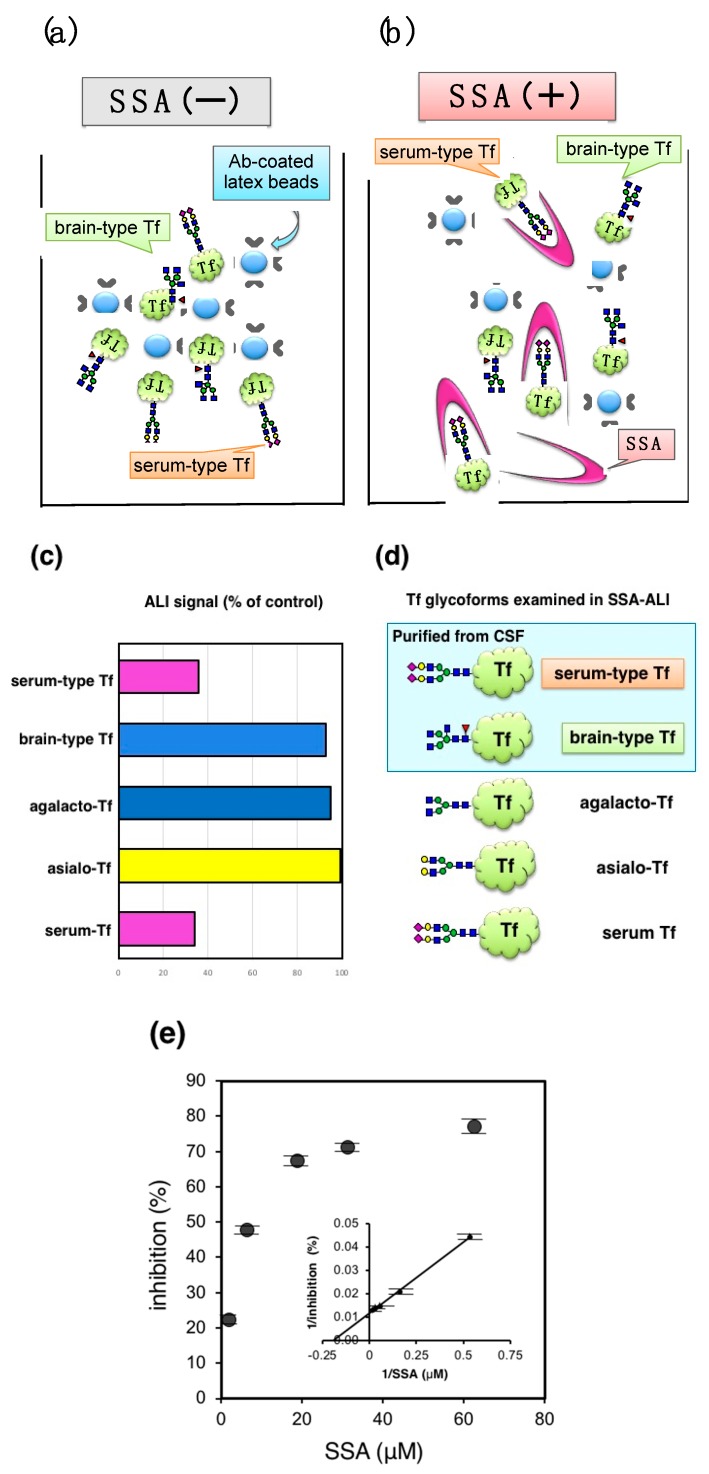
SSA lectin inhibition in latex-agglutination turbidimetric immunoassay. (**a**) Principle of latex-agglutination turbidimetric immunoassay (ALI) is illustrated; (**b**) SSA lectin inhibits the latex-agglutination; (**c**) ALI signals (%) relative to control (+BSA) are indicated; (**d**) Tfs examined are purified brain-type Tf, serum-type Tf, serum Tf, asialo-Tf, and agalacto-Tf; (**e**) Dose dependent inhibition of ALI signal for serum Tf (Hoshi et al., unpublished data).

Automated latex-agglutination turbidimetric immunoassay (ALI) is a rapid and high-throughput method for detecting various antigens including Tf. Indeed, several hundred assays are performed daily in hospital care, indicating that application of lectin inhibition to ALI would facilitate clinical use of glycoforms analysis. For detecting Tf in ALI, Tfs are mixed with Tf-Ab-coated latex particles to form aggregates that increase turbidity (Figure 5a). The concentration of Tf in the sample is quantified by measuring turbidity. The method is completed within 12–15 min in a liquid phase of a flow cell in an autoanalyzer LABOSPECT 003 (Hitachi High-Technologies Corporation, Tokyo, Japan), which is commonly used in hospitals. The addition of SSA was expected to inhibit the signal (Figure 5b) (SSA-ALI).

Schemas of Tf isoforms used for SSA-ALI are indicated in Figure 5d. SSA inhibits aggregate formation induced by serum-type Tf and serum Tf, but barely inhibits those of brain-type Tf, asialo-Tf, and agalacto-Tf (Figure 5c). The result indicates that SSA-ALI is a specific assay for α2,6-sialylated glycoforms of Tf. ALI signal with serum Tf was inhibited in a saturable manner. Reciprocal plot analysis revealed that the maximal inhibition is 77% and half maximal inhibition is observed at 8.4 μM of SSA (Figure 5e). For quantification of serum-type Tf by SSA-ALI, a standard curve was linear in a range of 1.25–20 μg/mL (*r* = 0.997). Dilution linearity of CSF was in the range of 0.125–0.50 μL. A recovery test reveals that 96–98% of added serum-type Tf in CSF was recovered. Day-to-day variation was less than 8% [10].

**Figure 6:**
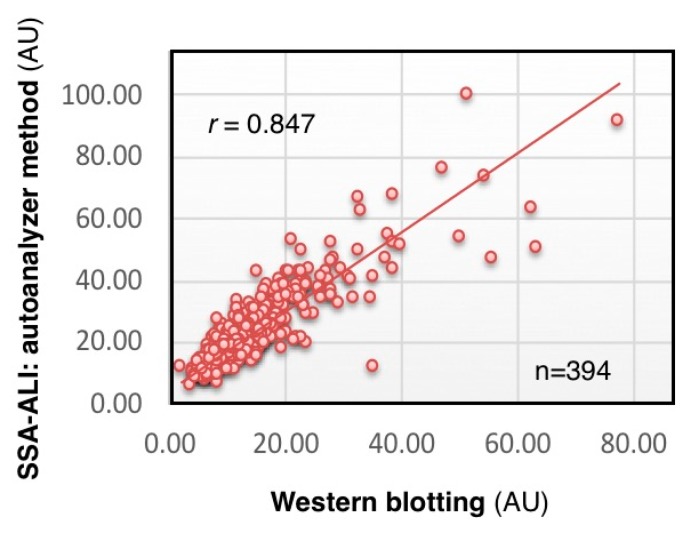
Correlation of SSA-ALI and Western blot results. Serum-type Tf levels in the CSF of 348 individuals are estimated by SSA-ALI and Western blotting (Hoshi et al., unpublished data).

We analyzed CSF concentration of serum-type Tf in 348 individuals by SSA-ALI and Western blotting. The results of SSA-ALI are correlated with those of Western blotting (*r* = 0.847, *p* < 0.001), indicating that SSA-ALI is useful for quantifying serum-type Tf as well as total Tf in CSF (Figure 6) (Hoshi et al., unpublished data). We are currently searching for a strong binder to brain-type Tf that would be applicable to ALI for a high-throughput assay for diagnosing iNPH.

## 4. Lectin Inhibition Assay in Immunohistochemistry

**Figure 7:**
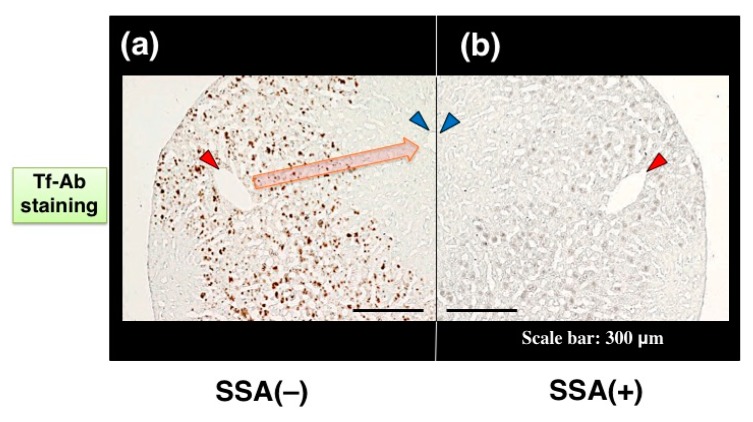

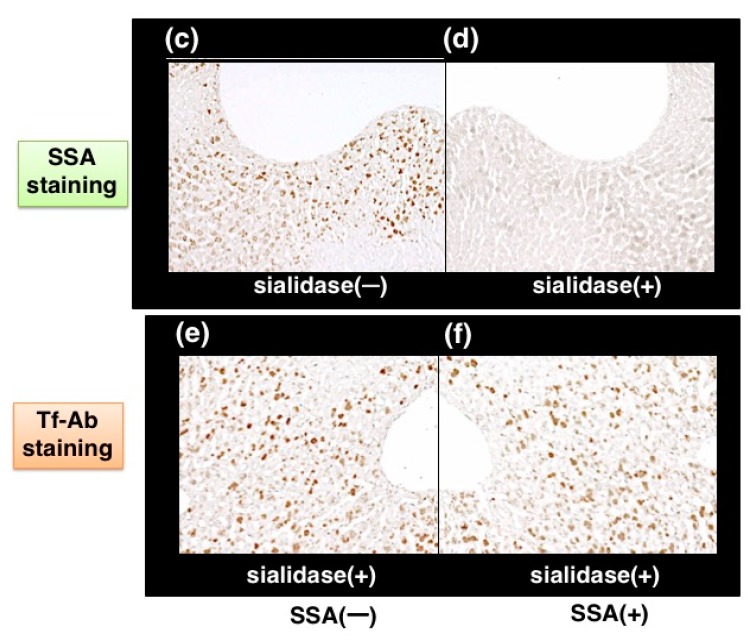
SSA inhibition in immunohistochemistry of liver section. A pair of mirror image sections of the liver are prepared: (**a**) the one is stained with Tf-Ab; (**b**) the other is stained in the presence of SSA. Blood flows from a interlobular vein (red arrowhead) into a central vein (blue arrowhead), as indicated with orange arrow (**a**). A pair of liver sections were pre-treated with (+) or without (−) sialidase and then subjected to SSA staining (**c**,**d**). Sialidase-treated liver sections were stained with Tf-Ab in the presence (+) or absence (−) of SSA (**e**,**f**). The data is reproduced with permission from Matsumoto et al., *J. Biochem.*, Oxford University Press, 2014 [11].

Visualizing a certain glycoform in tissue sections has been difficult. We tried to apply SSA inhibition to immunohistochemistry. Except the treatment with or without SSA, a pair of tissue sections on a single slide glass was stained with anti-Tf antibody in the same solution, minimizing staining difference between the sections. Human serum Tf is biosynthesized in the liver and then secreted into blood. Immunohistochemistry reveals that some hepatocytes near interlobular veins are stained by Tf-Ab (Figure 7a, red arrowhead) whereas hepatocytes near interlobular veins are hardly stained (Figure 7a, blue arrowhead). Pre-treatment of the liver section with SSA lectin significantly reduces Tf signals (Figure 7b), suggesting that SSA inhibits α2,6-sialylated-Tf signal in immunohistochemistry [11]. To examine the requirement of sialic acida residues for inhibition, a tissue section was treated with sialidase prior to analysis and removal of α2,6sialic acid residue was confirmed by SSA staining (Figure 7c,d). On the sialidase-treated section, SSA showed subtle effects on Tf-Ab staining (Figure 7e,f), suggesting that SSA binds to sialic acid residues on Tf in the liver section. It is notable that a pair of sialidase-treated sections, without SSA inhibition, show similar intensity of Tf-Ab staining, verifying the methodology for comparing a pair of sections (Figure 7e,f).

**Figure 8:**
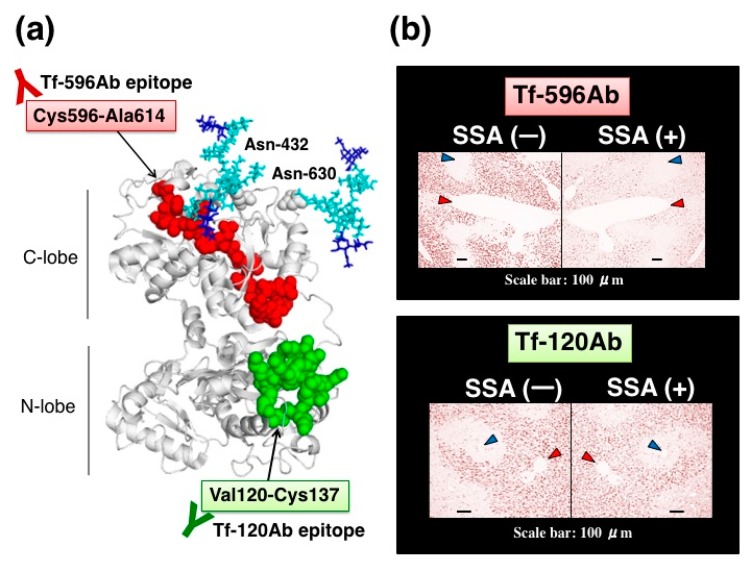
SSA inhibition using site-directed anti-peptide antibodies against Tf. (**a**) Localization of antigen epitopes for site-directed anti-peptide antibodies on a 3D-model of Tf. One antibody, Tf-596Ab, was prepared against a peptide sequence, Cys596-Ala614, (highlighted with red). The other antibody, Tf-120Ab, was prepared against a peptide sequence, Val120-Cys137, (highlighted with green). Asn-432 and Asn-630 are *N*-glycosylation sites. (**b**) A pair of mirror image sections is stained with Tf-596Ab (upper panel) or Tf-120Ab (lower panel) in the presence or absence of SSA. Interlobular and central veins are indicated with red and blue arrowhead, respectively. The data is reproduced with permission from Matsumoto et al., *J. Biochem.*, Oxford University Press, 2014 [11].

We further characterized SSA inhibition using site-directed anti-peptide antibodies for Tf. Tf has two *N*-glycosylation sites at Asn-432 and Asn-630 in its amino acid sequence (Figure 8a). These asparagine residues are mapped on the C-lobe at the carboxy-terminal domain and are close to each other on a 3D-model. We prepared two anti-peptide antibodies against Tf; one antibody (Tf-596Ab) was prepared against a peptide sequence, Cys596-Ala614, which was close to two *N*-glycosylation sites (Figure 8a, highlighted with red). The other antibody (Tf-120Ab) was prepared against a peptide sequence, Val120-Cys137, which is located far from *N*-glycosylation sites on the *N*-lobe (Figure 8a, highlighted with green). Tf-120Ab and Tf-596Ab were raised against chemically synthesized peptides with an expectation that these antibodies would recognize antigen peptide epitopes and react equally with various glycoforms of Tf. Indeed, like Tf-Ab, both Tf-120Ab and Tf-596Ab reacted with Tf in serum, purified α2,6-sialylated-Tf, and asialo-Tf on immunoblots, suggesting that Tf-120Ab and Tf-596Ab reacted with Tfs regardless of their glycoforms.

Anti-peptide antibodies are subjected to SSA inhibition assay in immunohistochemistry. In the absence of SSA, both anti-peptide antibodies stain some hepatocytes near interlobularveins, but not hepatocytes near central veins. In the presence of SSA, Tf-596Ab staining is markedly reduced (Figure 8b, upper right panel), whereas Tf-120Ab staining is subtly reduced by SSA (Figure 8b, lower right panel). Thus, SSA subtly inhibited Tf-120Ab, which specifically recognizes the peptide sequence distal to the glycosylation sites. In contrast, SSA remarkably inhibited Tf-596Ab recognizing the peptide sequence proximal to the glycosylation sites. These results suggest that proximity between the antigen epitopes and *N*-glycosylation sites is important for SSA inhibition [11].

## 5. Application of SSA Inhibition for Quantifying α2,6-sialylated CEA in Tissue Extracts

**Figure 9:**
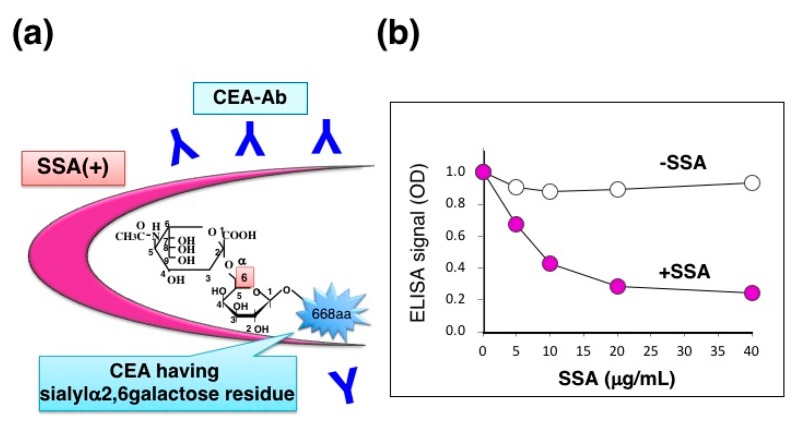
SSA inhibition for ELISA signal of CEA. (**a**) Schematic diagram of SSA binding to α2,6-sialylated-CEA; (**b**) Dose-dependent inhibition of CEA-ELISA signal by SSA in the concentration range of 0–40 μg/mL. The Figure 9b is adapted from Ito et al., *Proteomics.*, John Wiley & Sons, Inc., 2016 [12].

**Figure 10:**
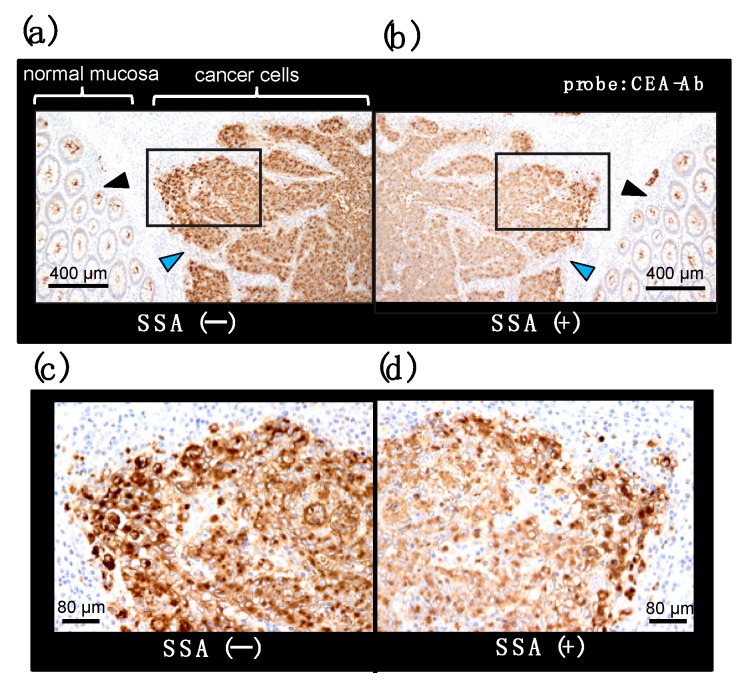
SSA inhibition in immunohistochemistry for CEA on a colon adenocarcinoma section. A pair of mirror image sections of a colon adenocarcinoma tissue is stained with an anti-CEA antibody in the presence (**b**) or absence (**a**) of SSA. Cancer cell and normal mucosa area are indicated with blue and black arrowhead, respectively. Squared portions are shown in a high magnification image (**c**,**d**) (Ito et al., unpublished data).

Carcinoembryonic antigen (CEA) is a glycoprotein marker that is widely used for diagnosing various cancers, especially colon adenocarcinoma. CEA carries α2,6-sialyl residues on its *N*-glycans whereas a normal counterpart, normal fecal antigen-2 (NFA-2), does not, suggesting that cancer-specific α2,6-sialylation on CEA may play a role in malignant phenotypes such as cell invasion and metastasis. SSA appears to bind α2,6-sialylated-CEA and inhibit its ELISA signal (Figure 10a). We therefore extracted CEA from formalin-fixed colon adenocarcinoma tissues by using PBS containing 1% NP-40 detergent. CEA concentration was determined by sandwich ELISA using two specific antibodies: CD66e Ab-2 (Cat. no. RB-368-A, Thermo Fisher Scientific, Inc., Waltham, MA, USA) and anti-human CEA (clone 1B2, Cat.no. 10094, Immuno-Biological Laboratories, Co., Ltd., Fujioka-Shi, Japan). The extracts contained CEA in a range of 1.40–3.92 μg/g tissue (*n* = 3). Indeed, detergent extracts showed a fair amount of ELISA signal in the absence of SSA whereas the signal was markedly reduced in the presence of SSA (Figure 9b), suggesting that the extracts contain α2,6-sialylated-CEA. SSA inhibition is dose-dependent and maximal inhibition level reaches to 74% in the concentration range of 20–40 μg/mL [12].

## 6. Lectin Inhibition for Immunohistochemistry of α2,6-sialylated-CEA

CEA is a member of the immunoglobulin (Ig) superfamily and, like other Ig-like molecules, it mediates aggregation of colonic adenocarcinoma cells, which appear to favor invasive nest formation. We examined localization of α2,6-sialylated-CEA in cancer invasion areas of colon adenocarcinoma tissue. Immunohistochemistry reveals infiltration of undifferentiated cancer cells (Figure 10a, blue arrowhead). Normal mucosa is also observed, in which apical portions are stained with CEA-Ab (Figure 10a, black arrowhead). Pre-treatment of the section with SSA lectin significantly reduced CEA signals in areas of infiltrative cancer (Figure 10b), suggesting the presence of α2,6-sialylated-CEA in the area. In contrast, signals in normal mucosa are not inhibited (Figure 10b). This is consistent with the observation that normal mucosa cells express NFA-2 in the apical portion. In high magnification, cytosol of cancer cells is heavily stained with CEA-Ab (Figure 10c). Pre-treatment of section with SSA attenuates CEA signals (Figure 10d) (Ito et al., unpublished data). Combination of histology and biochemistry with SSA inhibition will reveal the presence of α2,6-sialylated-CEA in cancer tissue, which is reported to be associated with liver metastasis [16]. The analysis would provide useful information for cancer treatment strategy; e.g., early intensive chemotherapy after surgery.

## 7. Conclusions

In this review, we demonstrate that SSA inhibits antibody binding to α2,6-sialylated-Tf and α2,6-sialylated-CEA in a glycoform-specific manner. The SSA inhibition is also applicable for visualizing the localization of α2,6-sialylated-Tf in the liver and α2,6-sialylated-CEA in colonic adenocarcinoma tissue. This is the first immunohistochemical demonstration of glycoform localization in a tissue section. SSA inhibition requires binding of SSA to sialylα2,6galactose epitopes of glycoproteins, because removal of sialic acid by sialidase treatment markedly attenuated SSA inhibition signals. SSA forms tetramer (130 kDa), the size of which may partly account for hindrance of many antigen epitopes on Tf (75 kDa). This speculation may be supported by the observation that RCA120 (120 kDa), PVL (40kDa), and AAL (28kDa) lectin inhibited Tf-Ab binding to asialo-Tf and brain-type Tf by 27, 13 and −2%, respectively (Figure 4). In addition to the lectin size, many other factors such as the number of glycans and the molecular size of antigens and relative affinities of lectins and antibodies would also affect the lectin inhibition. Systemic analysis using various lectins and antigens will be needed for further characterization of lection inhibition in the future.

Partial inhibition of antibody binding to an antigen was also reported for *Lens culinaris* agglutinin (LCA) lectin, recognizing core fucose (13). The lectin inhibited monoclonal antibody binding to fucosylated alpha-fetoprotein, an early marker of hepatocellular carcinoma (14, 15). However, LCA inhibited only 2 out of 30 monoclonal antibodies; one was inhibited by 36% and the other was 59%. Suzuki et al. reported that LCA binding to the core fucose sterically hinders neighboring antigen epitopes recognized by the two monoclonal antibodies. We demonstrated that SSA lectin inhibits polyclonal Tf-Ab binding, suggesting that binding complex of SSA and α2,6-sialylated glycans is flexible enough to hinder a substantial number of antigen epitopes on Tf. This speculation led us to examine SSA inhibition using site-directed peptide antibodies. One is Tf-596Ab against a peptide sequence, Cys596-Ala614, which was proximal to *N*-glycosylation sites. The other is Tf-120Ab against a peptide sequence, Val120-Cys137, which was distal to *N*-glycosylation sites. SSA inhibits binding of Tf-596Ab but affects Tf-120Ab only a little (Figure 8), suggesting that proximity of the antigen epitope to *N*-glycans is important for SSA inhibition. SSA complexed with the *N*-glycans may inhibit most polyclonal Tf-Ab, but it may not inhibit some antibody clones that recognize ‘distal’ antigen epitopes. This may explain why SSA partially inhibits polyclonal Tf-Ab signals in immunohistochemical and biochemical assays. Further analysis will be required for addressing the exact molecular mechanisms of lectin inhibition.

In conclusion, SSA lectin inhibition assay would be useful for detecting α2,6-sialylated glycoform of Tf and CEA even in crude specimens and tissue sections. The method would further be applicable to glycobiology and clinical medicine.
